# Direct crosstalk between GPCRs and ion channels via G proteins

**DOI:** 10.1038/s12276-025-01588-w

**Published:** 2025-11-16

**Authors:** Sun-Hong Kim, Jinhyeong Kim, Insuk So, Hyung Ho Lee

**Affiliations:** 1 Department of Chemistry, College of Natural Sciences, Seoul National University, Seoul, Republic of Korea; 2 Department of Physiology and Biomedical Sciences, Seoul National University College of Medicine, Seoul, Republic of Korea; 3 Institute of Human-Environment Interface Biology, Seoul National University, Seoul, Republic of Korea

**Keywords:** Biochemistry, Molecular biology

## Abstract

In recent years, cryo-electron microscopy structures of ion channels in complex with G proteins have been resolved, providing insights into the molecular mechanisms underlying the crosstalk between G protein-coupled receptors (GPCRs) and ion channels. Downstream signaling initiated by GPCR activation can indirectly modulate ion channel activity. Alternatively, the direct binding of Gα or Gβγ subunits to ion channels can directly regulate their ion conduction activity. Recent cryo-electron microscopy structures, such as TRPC5–Gα_i3_, GIRK–Gβγ and TRPM3–Gβγ, have elucidated these direct interactions and advanced our understanding of how Gα or Gβγ subunits activated by GPCRs modulate ion channel activity. In addition, the structure of the TRPV4–RhoA complex has revealed that small G proteins can also directly modulate ion channels. Understanding the physiological roles of these complexes will be critical for their potential use as pharmacological targets. Here we summarize the current knowledge of the interactions between ion channels and G proteins.

## Ion channels and G proteins reveal new signaling pathways

Ion channels are crucial for cell communication, allowing ions to move across cell membranes. This study explores how G proteins, which are molecules that help to transmit signals inside cells, interact with ion channels. The authors focus on a specific type of ion channel called transient receptor potential (TRP) channels, which are involved in sensing changes in the environment. This research highlights how G proteins, made up of subunits such as Gα and Gβγ, can directly bind to these ion channels and influence their activity. Using advanced imaging techniques such as cryo-electron microscopy, the authors examined the structures of these complexes to understand how they work. They found that G proteins can either activate or inhibit ion channels by binding to them directly. This interaction is important for various cellular processes and could be linked to certain diseases when it goes wrong.

This summary was initially drafted using artificial intelligence, then revised and fact-checked by the author.

## Introduction

Ion channels are essential components of cellular signaling, enabling the rapid and precise flux of ions across cellular membranes^1^. Their unique ability to gate ion-permeating pores in response to diverse extracellular and intracellular stimuli is crucial for maintaining cellular homeostasis^2^. This includes roles in nonexcitable cells, where they modulate signaling pathways that govern cell growth, differentiation and function^2^. The regulation of ion channels is controlled by a complex interplay of mechanisms, with G protein-coupled receptor (GPCR) signaling emerging as a key regulatory pathway^3^.

**Fig. 1:**
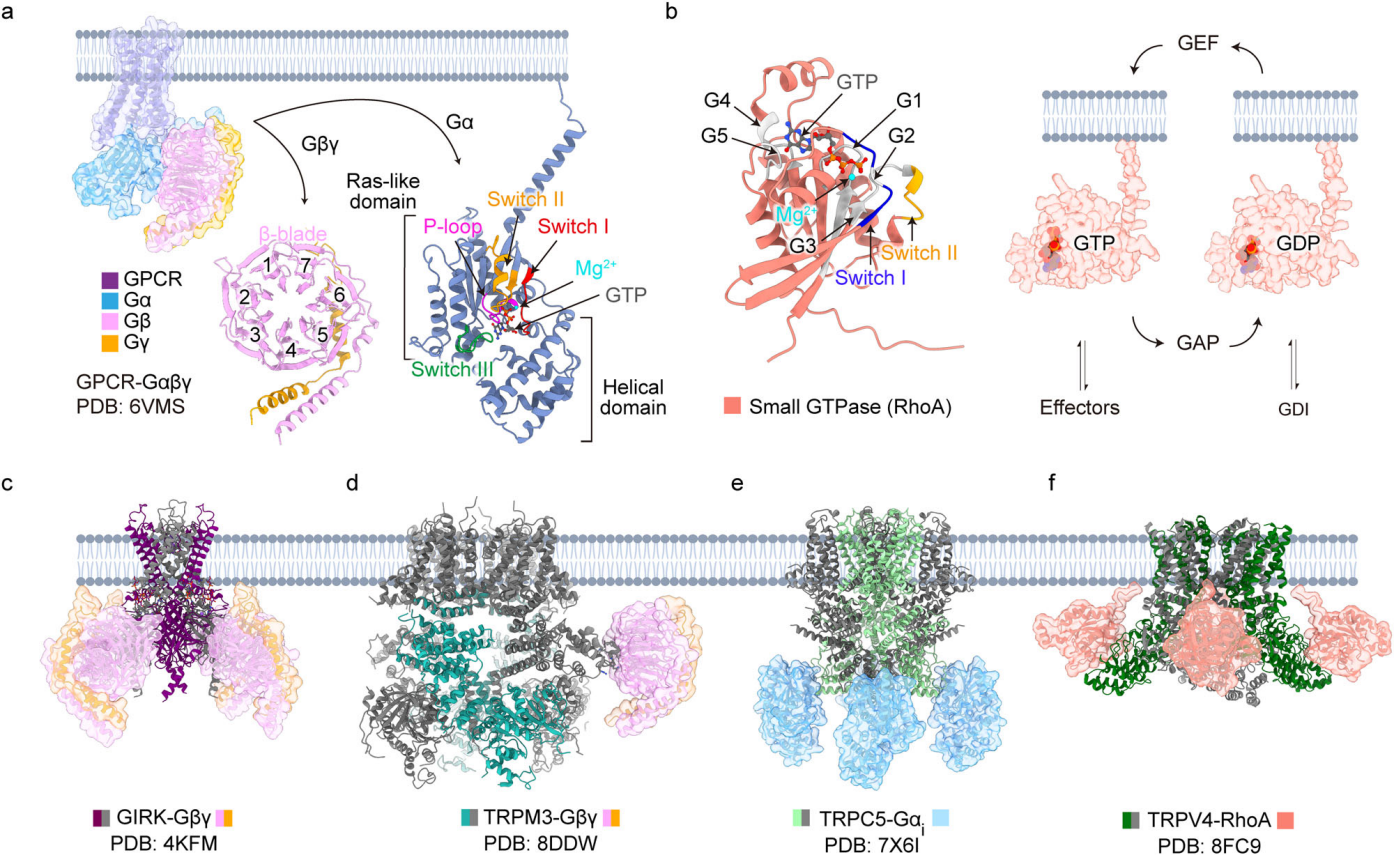
Complex structures of ion channels and G proteins. **a** Activation of GPCR (PDB, 6VMS) leads to the dissociation of the heterotrimeric G protein into the Gβγ dimer (pink and orange) and the Gα subunit (blue, AF-P08754-F1). The Gβγ dimer comprises a seven-bladed β-propeller structure, while the Gα subunit includes a helical domain and Ras-like domain containing critical elements such as Mg^2+^, GTP, the P-loop and switch I–III regions. **b** Structure of the small GTPase RhoA (salmon, AF-P61586-F1) with labeled functional elements: G1–G5, Mg^2+^ and switch I–II regions. The activation–inactivation cycle of GTPases is shown (right), mediated by GEFs for activation, GAPs for inactivation and GDIs for recycling. **c**–**f** The structures of ion channel complexes with G proteins or small GTPases: X-ray crystallography structure of the GIRK channel in complex with the Gβγ heterodimer (PDB, 4KFM) (**c**), cryo-EM structure of the TRPM3 channel in complex with the Gβγ heterodimer, tethered by an ALFA nanobody (PDB, 8DDW); the transmembrane region (residues 762–1,177) of TRPM3 in the presence of soluble Gβγ is aligned and visualized (PDB, 8DDQ) (**d**) cryo-EM structure of the TRPC5 channel in complex with the Gα_i3_ subunit (PDB, 7X6I) (**e**), cryo-EM structure of the TRPV4 channel in complex with small GTPase RhoA (PDB, 8FC9) (**f**).

Heterotrimeric G proteins are composed of three subunits: a Gα subunit (approximately 40 kDa), a Gβ subunit (37 kDa) and a Gγ subunit (8 kDa)^4^. In mammals, at least 16 α subunits, 5 β subunits and 11 γ subunits have been identified. The Gα subunit functions as a molecular switch, transitioning between active and inactive states on the basis of its nucleotide binding—guanosine 5′-triphosphate (GTP) or guanosine 5′-diphosphate (GDP)^3,5,6^. In the GDP-bound state, the Gα subunit forms a high-affinity complex with Gβγ and GPCRs. Upon external stimulation, the receptor triggers a conformational change that releases GDP and facilitates GTP binding, thereby disrupting the complex^7^ (Fig. 1a). The dissociated Gα and Gβγ subunits can remain anchored to the plasma membrane through the lipidation of Gα and Gγ^8^. They then diffuse across the plasma membrane and cytoplasm, transmitting cellular signals by interacting with effector proteins^9–11^. Later, this signaling is diversely terminated by the intrinsic GTPase activity of Gα, GPCR kinases (GRKs) and arrestins, which are multifunctional adapter proteins with diverse roles, including their own signaling^12,13^.

The crosstalk between G proteins and ion channels represents a complex and nuanced signaling mechanism, broadly categorized into direct and indirect regulation. Indirect regulation, which is prominent in vivo, typically involves small secondary messengers such as calcium (Ca^2+^) and cyclic adenosine monophosphate^14^, which indirectly influence the activity of ion channels. Alternatively, ion channels can be directly regulated by the binding of Gα or Gβγ subunits to the channels, following their release from GPCRs in response to external stimuli that activate the receptors.

In recent years, cryo-electron microscopy (cryo-EM) structures of ion channels in complex with G proteins have been resolved, with the majority involving transient receptor potential (TRP) channels (Fig. 1c–f). TRP channels, which are nonselective cation channels, facilitate the permeation of various cations, including calcium—a pivotal mechanism for modulating cell signaling in nonexcitable cells^15^. Widely recognized as polymodal cellular sensors expressed across various tissues, TRP channels are increasingly identified as effector molecules of Gα or Gβγ subunits. In addition, small G proteins, such as Ras, which are structurally related to the α subunit of heterotrimeric G proteins, have been shown to directly bind to TRP channels^16–18^.

As recent cryo-EM structures of TRP channels have been extensively reviewed elsewhere^19–22^, this review focuses on the currently available G protein–ion channel complexes, with particular emphasis on G protein–TRP channel complexes (Table 1). In addition, direct interactions between ion channels and G proteins, although not yet structurally characterized but supported by biochemical and cell biological evidence, are summarized (Table 2). Overall, this review aims to explore the molecular mechanisms of crosstalk between GPCRs and ion channels, focusing on the direct binding of Gα or Gβγ subunits to ion channels.

**Table 1:** List of structures of G protein subunits with ion channels.

G protein		Ion channel	Regulation^a^	Affinity^b^	State	PDB ID	Ligands	Reference
Gα	Gα_i3_	TRPC5	↑	*K*_d_ = 0.91 μM	Closed	7X6I	–	^79^
	Gα_i3_	TRPC5	↑		Closed	8GVX		
Gβγ	Gβ1γ2	GIRK2 (Kir3.2)	↑	*K*_d_ = 1,900 μM (first) to 50 μM (fourth)	Closed	4KFM		^50,56^
	Gβ1γ2	TRPM3 peptide	↓	*K*_d_ = 56 μM	–	6RMV		^51^
	Gβ1γ2	TRPM3	↓	IC_50_ = 0.24 μM	Closed	8DDQ		^52^
	Gβ1γ2	TRPM3	↓		Closed	8DDW		
	Gβ1γ2	TRPM3	↓		Closed	8DDX	PIP_2_	
Small GTPase	RhoA	TRPV4	↓	–	Closed	8FC9	–	^16^
	RhoA	TRPV4	↓		Closed	8FC7	GSK279	
	RhoA	TRPV4	↓		Open	8FCB	GSK101	
	RhoA	TRPV4	↓		–	8T1C	–	^17^
	RhoA	TRPV4	↓		Closed	8JVJ	–	^18^

^a^Arrows in the table indicate either up- or downregulation of ion channel activities by G proteins in each case.

^b^Binding affinities (*K*_d_ values) were measured using direct binding assays. Representative examples include TRPC5–Gα_i3_ (excised inside-out patch), GIRK2 (Kir3.2)–Gβ1γ2 (isothermal titration calorimetry) and TRPM3 peptide–Gβ1γ2 (bio-layer interferometry, BLI). By contrast, the IC_50_ value, which indicates the concentration required to inhibit channel activity by 50%, was derived from functional assays, for instance, TRPM3–Gβ1γ2 was measured using an excised inside-out patch.

**Table 2:** Direct regulation of ion channels by G proteins.

G protein		Ion channel	Regulation^a^	Direct binding^b^	Functional test^b^	Direct recording^b^	X-ray crystallography	Cryo-EM
				[Reference]				
Heterotrimeric G protein	Gβγ	GIRK2	↑		Whole-cell patch clamp/oocyte^53^	Lipid bilayer/*Pichia pastoris*^55,56^	^50^	
		TRPM3	↓	Co-IP/HEK293^51,68,69^, BLI, dot blot analysis/Sf9, *Escherichia coli*^51^	Whole-cell patch clamp/HEK293^51,52,68–70^/oocyte^53^/DRG neuron^68–70^ Two-electrode voltage clamp/oocyte^51,68^ Calcium imaging/HEK293^68,69^/DRG neuron^68–70^	Inside-out patch clamp/HEK293^52,70^, /oocyte^68^	^51^	^52^
		TRPM1	↓	Co-IP/HEK293^72^	Whole-cell patch clamp/retinal bipolar cell^72^, HEM, HEK293^75^ Calcium imaging/HEM, HEK293^75^			
		Ca_V_2.2	↓		Whole-cell patch clamp/sympathic neuron^76^ /DRG neuron^77^			
	Gα_i3_	TRPC5	↑	Co-IP/HEK293^79^	Whole-cell patch clamp/HEK293^79,87^	Inside-out patch clamp/HEK293^79^		^79^
	Gα_i2_	TRPC4	↑	Co-IP/HEK293^87,95^, pull-down assay/*E. Coli*^87^	Whole-cell patch clamp/HEK293^87,95^ Calcium imaging/HEK293^95^			
	Gα_q_	TRPC1/4 TRPC1/5	↑	Co-IP/HEK293^98^	Whole-cell patch clamp/HEK293^98^			
	Gα_q_	TRPM8	↓	Co-IP, pull-down assay/HEK293, DRG neuron^99^ Pull-down assay/HEK293^100^	Whole-cell patch clamp/HEK293^99,100^	Inside-out patch clamp/HEK293^99^		
	Gα_o_	TRPM1	↓	Co-IP/HEK293^72^	Whole-cell patch clamp/retinal bipolar cell^72^			
	Gα_o_	GIRK1/GIRK2	↑	Co-IP/rat brain tissue Pull-down assay/*E. Coli*^104^		Inside-out patch clamp/pyramidal cell^103^		
	Gα_s_	Ca_V_1.2	↑			lipid bilayer/cardiac sarcolemmal vesicle^105^ /skeletal muscle T-tubule^106,107^		
	Gα_s_	Na_V_1.5	↓			Inside-out patch clamp/ventricular myocyte^108^		
Small G protein	RhoA	TRPV4	↓	Co-IP, co-incubation, pull-down assay/HEK293, MN1 NMR spectroscopy/*E. Coli*^113^	Calcium imaging, FRET assay/stable cell line^113^			^16–18^
	Rab11a	TRPV5/TRPV6	↓	Yeast two-hybrid, Co-IP, pull-down assay/oocyte, *E. Coli*^115^				
	RGKs	Ca_V_1/Ca_V_2	↓	Co-IP, pull-down assay/COS-1, HEK293^119,127^, co-IP/HEK293^120,123,124,126^, Yeast two-hybrid, co-IP, pull-down assay/HEK293^122^	Whole-cell patch clamp, FRET assay/HEK293, tsA201^120,127^ Whole-cell patch clamp, calcium imaging/PC12, MIN6, BHK, oocyte^122,126^ Whole-cell patch clamp/HEK293, HIT-T15, ventricular myocyte^123–125^			

^a^Arrows in the table indicate either up- or downregulation of ion channel activities by G proteins in each case.

^b^References for direct binding include co-immunoprecipitation (co-IP), BLI and binding affinity assays such as pulldown and yeast two hybrid, among other techniques. References for functional tests using expression systems include whole-cell patch-clamp recordings, Ca^2+^ imaging and Förster resonance energy transfer (FRET) assays. References for direct recordings include data from inside-out patch-clamp recordings and lipid bilayer systems. DRG, dorsal root ganglion.

## Structural features of heteromeric and small G proteins

Before exploring the role of G protein subunits in the direct regulation of ion channels, it is helpful to first review their well-characterized structural features. The Gα subunit consists of a Ras-like GTPase domain and a helical domain, with the guanine nucleotide-binding site positioned between them^23^ (Fig. 1a). The Ras-like GTPase domain comprises six-stranded β-sheets surrounded by five α-helices^24,25^ and contains conserved GTP-binding motifs, including the phosphate-binding loop (P-loop), Mg^2+^ binding residues and two guanine ring-binding sites^3,26^. The three switch regions (switch I–III) undergo conformational changes depending on the phosphate state of the bound guanosine nucleotide. Specifically, GTP binding induces a tighter packing of switch I and switch II, driven by the presence of an additional phosphate group^23,27^.

The Gβ and Gγ subunits form a stable heterodimeric complex that associates with Gα to assemble the heterotrimeric G protein complex^23^. The Gβ subunit features an N-terminal α-helix and a propeller-like structure composed of seven motifs, known as β-blades^28^, each containing four-stranded antiparallel sheets. By contrast, the Gγ subunit is smaller and less stable than Gβ, forming helical domains that interact with the amino terminus of Gβ, which extends outward from the Gβ core. While the Gβ subunit is highly conserved, variations in the Gγ subunit and its prenylation at the carboxy terminus suggest that the functional specificity of the Gβγ complex may largely depend on the Gγ subunit^29,30^. In addition, Gβ stabilizes the N-terminal domain of Gα by directly binding to the α2-helix and interacting with the switch II region within the core^31^. Notably, unlike the Gα subunit, the Gβγ subunit undergoes minimal structural changes during signaling.

**Fig. 2:**
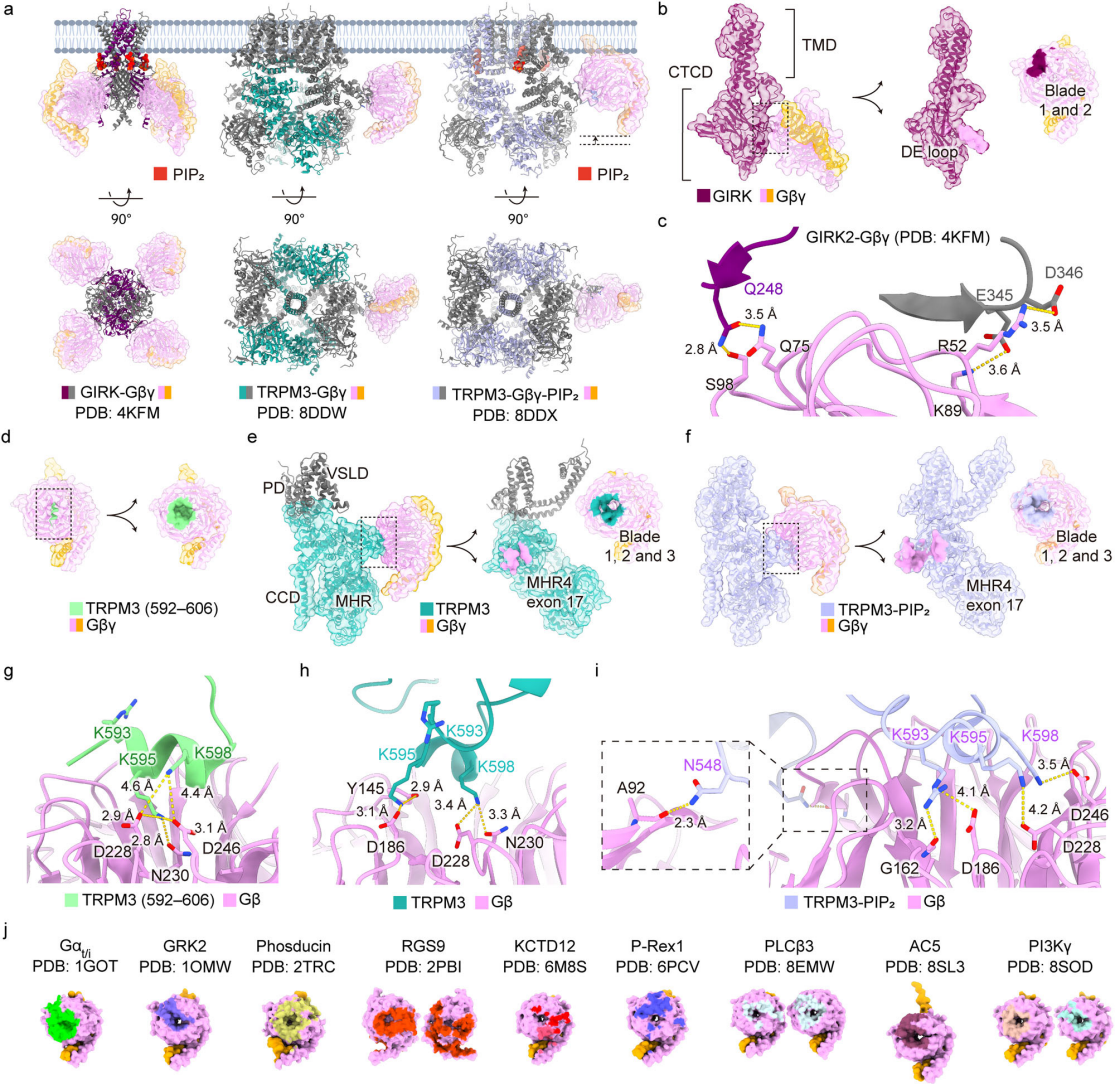
Gβγ in complex with ion channels and their binding interfaces. **a** Side and bottom views of ion channel complexes with Gβγ. From left to right: GIRK2–Gβγ (PDB, 4KFM), TRPM3–Gβγ (PDB, 8DDW) and TRPM3–Gβγ–PIP_2_ (PDB, 8DDX). **b** A detailed view of the GIRK2 protomer in complex with Gβγ, showing the TMD and CTCD. The binding interfaces include the DE loop of GIRK protomer and blades 1 and 2 of Gβ. **c** Hydrogen bonding between GIRK2 (purple) and Gβ (pink). Key residues include Q248 (GIRK) interacting with Q75 and S98 (Gβ). In addition, adjacent GIRK protomer (gray) residues E345 and D346 electrostatically interact with R52 and K98 (Gβ). **d** The crystal structure of the TRPM3 peptide (residues 592–606) in complex with Gβγ (PDB, 6RMV), with the binding interface highlighted. **e** Structural details of the TRPM3 channel in complex with Gβγ, including key domains: CCD, MHR and voltage-sensor-like domain (VSLD). The binding interface includes exon 17 of MHR4 (TRPM3) with blade 1, 2 and 3 (Gβ). **f** The TRPM3–PIP_2_–Gβγ complex (PDB, 8DDX) showing a similar interaction to that described in **e**. **g** Electrostatic and hydrogen bonding interactions in the TRPM3 peptide–Gβγ crystal structure (PDB, 6RMV). Key residues include K595 and K598 (TRPM3) with D228, N230 and D246 of the Gβ. Distances between residues are indicated. **h** Electrostatic and hydrogen bonding interactions in the cryo-EM structure of TRPM3–Gβγ (PDB, 8DDW). Differences from the crystal structure include K595 interacting with Y145 and D186, while K598 interacts with D228 and N230. **i** Altered interactions in the TRPM3–Gβγ–PIP_2_ complex (PDB, 8DDX), with additional hydrogen bonding (N548 and A92) highlighted in the dotted box. **j** Comparative analysis of Gβγ binding interfaces in various complexes, including Gα_t/i_ (PDB, 1GOT), G protein-coupled receptor kinases 2 (GRK2; PDB, 1OMW), phosducin (PDB, 2TRC), regulators of G protein signaling 9 (RGS9; PDB, 2PBI), KCTD12 (PDB, 6M8S), phosphatidylinositol 3,4,5-triphosphate-dependent Rac exchanger 1 (P-Rex1; PDB, 6PCV), phospholipase C-β3 (PLCβ3; PDB, 8EMW), adenylyl cyclase 5 (AC5; PDB, 8SL3) and phosphoinositide 3-kinase γ (PI3Kγ; PDB, 8SOD). The binding interfaces are highlighted in different colors.

**Fig. 3:**
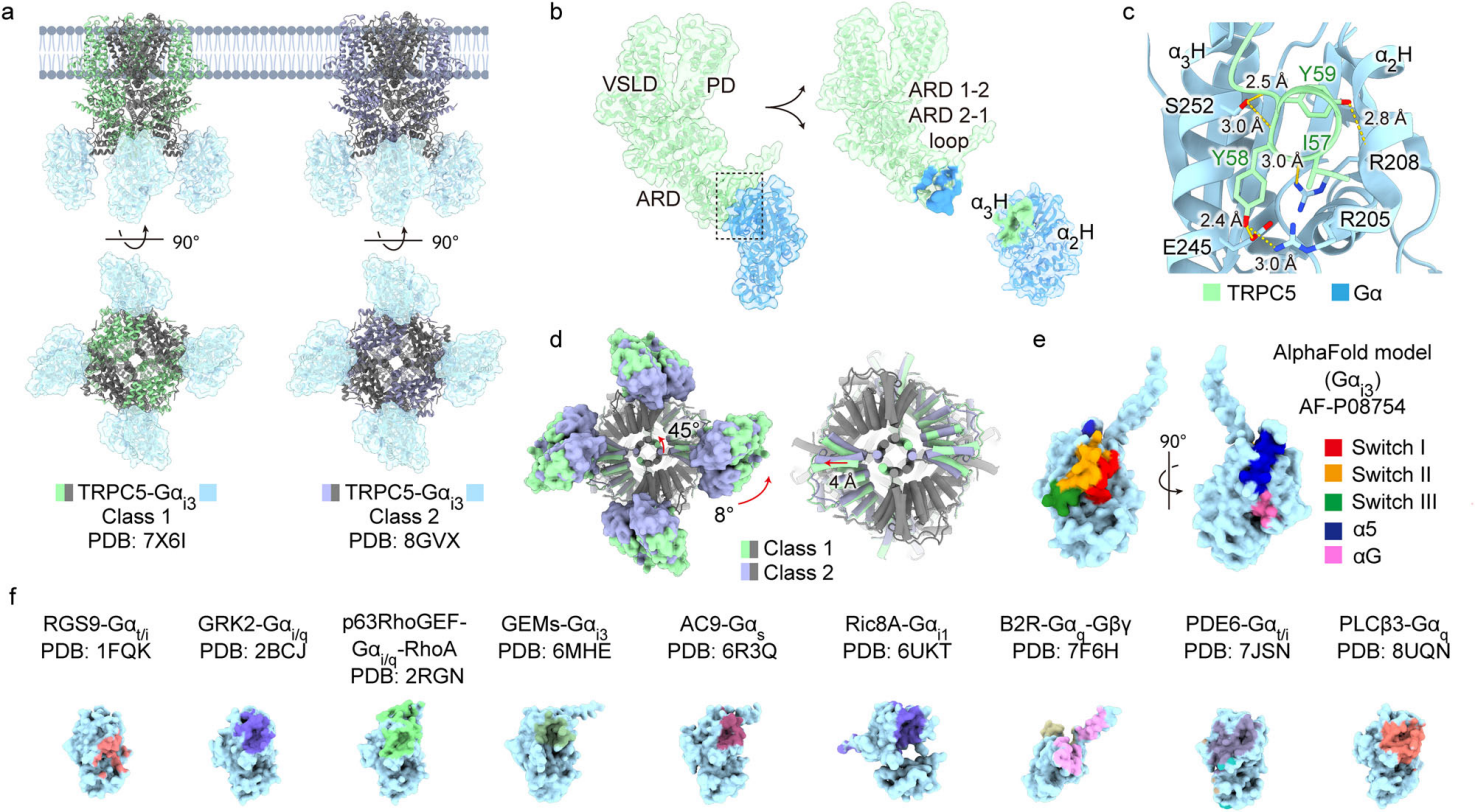
Gα_i3_ in complex with TRPC5 ion channels. **a** Cryo-EM structures of TRPC5 in complex with Gα_i3_, illustrating two different classes: class 1 (green; PDB, 7X6I) and class 2 (purple; PDB, 8GVX). Side and bottom views highlight structural differences between the classes. **b** Single TRPC5 protomer and Gα_i3_ subunit in class 1. The TRPC5 includes key domains: the VSLD, pore domain (PD) and ARD. The binding interfaces involve the loop connecting ARD 1-2 and 2-1 (TRPC5) with the cleft of the α2 and α3 helices (Gα_i3_). **c** A detailed view of the IYY motif in the loop. Residues I57, Y58 and Y59 of TRPC5 fit into the cleft between the α2 and α3 helices of Gα_i3_, forming hydrogen bonds with residues R205, R208 and E245. Interatomic distances are labeled in angstroms. **d** The variance between the two classes viewed from the bottom. Class 2 exhibits a 45° rotation in the CCD of TRPC5, an 8° rotation of Gα_i3_ and a 4-Å outward shift of the cytosolic ARD. These conformational changes are depicted with overlays of the two classes, highlighting differences in their relative orientations. On the right, G protein densities were removed to enhance visualization of the ARD. **e** AlphaFold-predicted structure of Gα_i3_ (AF-P08754) with regions color coded to indicate functional domains, including switch I–III and α5 and αG helices. **f** Comparative analysis of the Gα protein binding interfaces across various available complex structures. Examples include regulators of G protein signaling 9 (RGS9)–Gα_i/t_ (PDB, 1FQK), G protein-coupled receptor kinase 2 (GRK2)–Gα_i/q_ (PDB, 2BCJ), p63RhoGEF–Gα_i/q_–RhoA (PDB, 2RGN), guanine-nucleotide exchange modulators (GEMs)–Gα_i3_ (PDB, 6MHE), adenylyl cyclase 9 (AC9)–Gα_s_ (PDB, 6R3Q), resistance to inhibitors of cholinesterase 8A (Ric8A)–Gα_i1_ (PDB, 6UKT), bradykinin 2 receptor (B2R)–Gα_q_–Gβγ (PDB, 7F6H), phosphodiesterase 6 (PDE6)–Gα_t/i_ (PDB, 7JSN) and phospholipase C-β3 (PLCβ3)–Gα_q_ (PDB, 8UQN).

The Gβγ subunit interacts with multiple binding partners, as demonstrated by several resolved complex structures^32–37^, including G protein-coupled inwardly rectifying potassium channels (GIRK), phosphoinositide 3-kinase γ (PI3Kγ), phospholipase C (PLC) β and adenylyl cyclase (AC)—all of which are activated by Gβγ^38,39^ (Fig. 2j). Likewise, the Gα subunit binds to various effectors to propagate signals, many of which have been well characterized through structural analyses^40–46^. Recent cryo-EM structures have captured Gα in complex with AC, cGMP phosphodiesterase, GRK, resistance to inhibitors of cholinesterase (Ric) 8 and PLCβ (Fig. 3f).

**Fig. 4:**
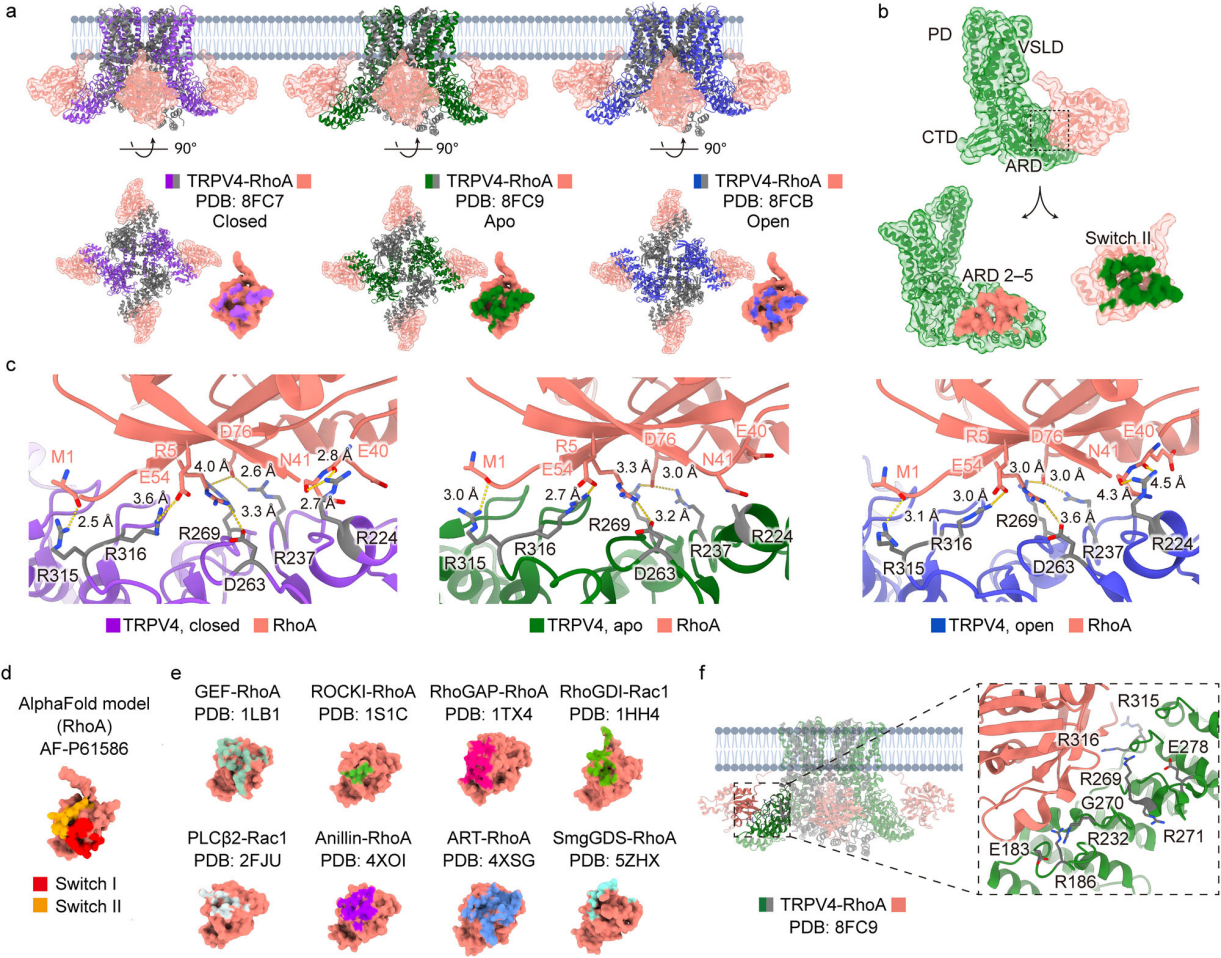
Small GTPase RhoA in complex with TRPV4. **a** Cryo-EM structures of TRPV4 in complex with small GTPases RhoA in three different states: antagonist-bound closed (purple; PDB, 8FC7), apo (green; PDB, 8FC9) and agonist-bound open (blue; PDB, 8FCB). Side and bottom views highlight changes in the RhoA binding interface across states. The apo state exhibits the largest binding interface, while the closed and open states show reduced contact. **b** Single TRPV4 protomer with key domains annotated: PD, VSLD, cytoplasmic terminal domain (CTD) and ARD. The binding interfaces are ARD 2–5 of TRPV4 and the switch II region of RhoA. **c** Binding interfaces between TRPV4 and RhoA in the closed (purple), apo (green) and open (blue) states. The interacting residues in TRPV4 are colored gray. In the closed state, residues E40 and N41 (RhoA) interact with R224 (TRPV4), an interaction that is maintained in the open state but at a greater distance. The interaction is not applicable in the apo state owing to the unmodeled side chain. Other key interactions include R237, D263, R269, R315 and R316 (TRPV4), with interatomic distances noted in angstroms. **d** AlphaFold-predicted structure of RhoA (AF-P61586), with functional regions switch I and switch II color coded to indicate key interaction sites. **e** Comparative analysis of small GTPase binding interfaces across other available structures. Examples include GEF–RhoA (PDB, 1LB1), ROCKI–RhoA (PDB, 1S1C), rho GTPase-activating protein (RhoGAP)–RhoA (PDB, 1TX4), rho guanine nucleotide dissociation inhibitors (RhoGDI)–Rac1 (PDB, 1HH4), phospholipase C-β2 (PLCβ2)–Rac1 (PDB, 2FJU), anillin–RhoA (PDB, 4XOI), ADP-ribosyltransferase (ART)–RhoA (PDB, 4XSG) and smgGDS–RhoA (PDB, 5ZHX). **f** Disease-related mutations in the N-terminal ARD region of the TRPV4 channel are displayed in gray.

Notably, small G proteins, which are structurally related to Gα (Figs. 1b and 4d), also directly bind to ion channels. Their interactions with effectors have been extensively characterized through structural studies (Fig. 4e), providing diverse insights into how small G proteins engage with their target molecules depending on their conformational states in the GTP- or GDP-bound forms. The conformational states of small G proteins are regulated by guanine nucleotide exchange factors (GEFs), GTPase-activating proteins (GAPs) and guanine nucleotide dissociation inhibitors (GDIs). Specifically, GEFs facilitate GDP release by sterically displacing switch I^47^, while GAPs stabilize the GTPase motif at the activation site to accelerate GTP hydrolysis^48^. GDIs, however, sequester key lipid modifications that are necessary for membrane anchoring, effectively preventing small G proteins from remaining in their active state at the plasma membrane^49^. The direct binding of small G proteins to ion channels will be discussed in a later section.

## Direct binding of Gβγ to ion channels

As a groundbreaking structural insight into the interaction between G proteins and ion channels, the structures of the GIRK2–Gβγ^50^ and TRP melastatin (TRPM) 3–Gβγ^51,52^ complexes were elucidated, revealing intriguing mechanisms by which Gβγ binds to and modulates ion channels (Fig. 2a). The distinct binding site of Gβγ on two different ion channels further highlights its multifaceted role beyond interactions with downstream effector proteins^53^.

### GIRK2–Gβγ complex

GIRK2 plays a crucial role in regulating cardiac and neuronal cells^54^, and owing to its vital function in survival, it has been extensively studied in the context of multi-ligand regulation^55^. Notably, phosphatidylinositol 4,5-bisphosphate (PIP_2_), Na^+^ and Gβγ each partially activate the channel individually, while their combined action results in greater activation^56–60^. Therefore, the contribution of Gβγ to GIRK2 activation must be evaluated alongside the binding of PIP_2_ and Na^+^.

The complex structure consists of one GIRK2 homotetramer, four Gβγ subunits, four PIP_2_ molecules and four Na^+^ ions bound to their respective regulatory sites (Protein Data Bank (PDB), 4KFM)^50^ (Fig. 2a). GIRK channels form tetrameric assemblies, comprising membrane-embedded transmembrane domains (TMDs) and cytoplasmic channel domains (CTCDs), where Gβγ binds at the interface between two adjacent CTCD subunits (Fig. 2b). Previous crystal structures, including wild type GIRK and the R201A mutant (a constitutively open variant that does not require G protein activation), in the presence or absence of PIP_2_, have provided valuable insights into the role of Gβγ in the activation of GIRK2^60^.

Mechanistically, the binding of Gβγ triggers a conformational shift, leading to the rotation of CTCDs relative to the TMDs and partial splaying of the inner helices—an intermediate state between the closed and fully open (R201A) conformations. Complete channel opening follows further CTCD rotation and an expanded splaying of the inner helical gate. Wang et al. proposed that the binding affinity between GIRK2 and Gβγ exhibits positive cooperativity^50,56^, probably driven by an allosteric rotation of the CTCDs, supported by structural evidence of Gβγ binding at adjacent protomers (Fig. 2c).

The activation of GIRK by Gβγ can be desensitized through the binding of Gβγ to the potassium channel tetramerization domain (KCTD), an auxiliary subunit of the inhibitory metabotropic gamma-aminobutyric acid (GABA_B_) receptor^61,62^. The crystal structure of the H1 domain of KCTD12 in complex with Gβγ (PDB, 6M8S) proposed a model for GIRK channel desensitization^35^. Initially, KCTD is bound to the distal cytoplasmic C terminus of the GABA_B_ receptor. Upon GABA_B_ receptor activation, Gβγ diffuses to activate the GIRK channel. However, owing to the higher binding affinity of Gβγ to KCTD compared to Gβγ to GIRK, when KCTD encounters the GIRK–Gβγ complex, it effectively sequesters Gβγ away from GIRK, leading to channel desensitization^35,61^. The high binding affinity of KCTD to Gβγ is attributed to its tight pentameric interaction, which involves cooperative Gβ–Gβ interactions. Taken together, direct binding of Gβγ to either GIRK or KCTD plays a crucial role in modulating GIRK channel activation and its desensitization.

### TRPM3–Gβγ complex

Given that GIRK and TRP channels share structural similarities as tetrameric cation channels with several conserved domains, it is particularly intriguing to explore the activation mechanism of TRP channels by Gβγ and compare its regulatory role in TRP channels with that in GIRK channels. The first structural evidence of TRP channel regulation by Gβγ emerged from studies on the TRPM3 channel. TRPM3 was previously known to be regulated by pregnenolone sulfate^63–65^, sphingosine^66^ and heat^65,67^. Later, the discovery of direct binding between TRPM3 and Gβγ introduced a novel regulatory mechanism^68–70^. Before this finding, because PIP_2_ is a prerequisite for TRPM3 function, the activation of Gβγ or Gα_q_ was thought to indirectly inhibit most TRP channels by triggering PIP_2_ hydrolysis via PLCβ^71^.

A total of 3 years after the direct regulation of TRPM3 by Gβγ was first proposed, the crystal structure of Gβγ bound to a TRPM3 peptide was elucidated (PDB, 6RMV)^51^ (Fig. 2d). This study identified exon 17 (residues 591–603) of TRPM3 as a Gβ binding site, which is absent in certain splicing variants. Subsequently, several cryo-EM structures of full-length TRPM3 (splice variant α2, residues 1–1,344) in complex with Gβγ were determined in a detergent environment^52^ (Fig. 2a). The previously determined crystal structure of TRPM3 in complex with a Gβγ peptide aligned well with the full-length TRPM3–Gβγ complex (PDB, 8DDW), revealing only slight differences in electrostatic interactions^51^ (Fig. 2g, h).

A notable difference was observed in the PIP_2_-bound structure of the TRPM3–Gβγ complex (PDB, 8DDX) compared to its PIP_2_-free form (Fig. 2e,f). In the presence of PIP_2_, the structure showed an upward movement of Gβγ toward the plasma membrane (Fig. 2a). Three key features of this movement are worth highlighting. First, a lipidation-like anchor of the Gγ subunit was observed, despite the use of a soluble C68S mutant for the study. Second, an additional contact between N548 of TRPM3 and A92 of Gβ was detected, as shown in the boxed region of Fig. 2i. Third, along with this additional contact, K598 moved closer to D246, K595 lost its previous interactions and K593 shifted toward Gβ’s G162 and D186 (Fig. 2i). Mutagenesis of K595 to alanine (K595A) weakened Gβγ-mediated inhibition, emphasizing the critical role of this residue in the regulatory mechanism.

However, the precise mechanism by which Gβγ binding to the melastatin homology region (MHR) domain of TRPM3 influences the channel pore remains unclear, as no noticeable structural changes were observed in the pore region. This lack of pore alteration may be explained by the observation that only a single Gβγ subunit was visible in the cryo-EM structure, rather than the expected 1:1 stoichiometry with each TRPM3 protomer, as seen in the GIRK2–Gβγ complex. This was the case even under conditions with a 400-fold (100 μM) excess of Gβγ (IC_50_ of 0.24 μM) and the use of the ALFA-nanobody tethering system to enhance Gβγ binding. Despite these uncertainties, the findings provide valuable insights into the regulation of ion channels by Gβγ, laying the foundation for understanding the crosstalk between two major classes of transmembrane proteins: GPCRs and ion channels.

### Gβγ binding to other ion channels

Although the structural evidence has yet to be elucidated, TRPM1 is another TRPM channel directly regulated by Gβγ^72^, sharing a high sequence identity (56%) with TRPM3, including the conserved region for Gβγ binding. A notable difference lies in their expression patterns: TRPM3 is highly expressed in the nervous system^73^, whereas TRPM1 is predominantly expressed in ON bipolar cells, where GPCRs such as metabotropic glutamate receptor 6 (mGluR6) and GPR179 are located^72,74^. Upon mGluR6 activation by glutamate, Gα_o_ and Gβγ are released from mGluR6, leading to the closure of TRPM1. However, the individual or combinatorial effects of each G protein subunit on TRPM1 remain a topic of debate^72,75^. Co-immunoprecipitation and bioluminescence resonance energy transfer assays have shown that both the N and C termini of TRPM1 bind to Gα_o_, while its N terminus binds to Gβγ^72^. Further structural studies are necessary to fully understand these interactions.

Other ion channels directly regulated by Gβγ include members of the voltage-gated calcium channel (Ca_V_) 2^76^. In the Ca_V_2.1 (P and Q type) and Ca_V_2.2 (N type) channels, the large pore-forming α subunit is regulated through direct binding with Gβγ^77^. The proposed Gβγ binding sites on Ca_V_2 channels are the intracellular connecting loops between domains I and II. However, other studies suggest that the N- and C-terminal tails serve as alternative binding sites^78^. Further structural studies are necessary to elucidate the overall architecture of the Ca_V_–Gβγ complex.

## Direct binding of Gα to ion channels

The direct binding of the Gα subunit to ion channels has been primarily studied in TRP channels. However, it is believed that TRP channels are not the only family of ion channels directly modulated by Gα, warranting further investigation in future studies. Currently, the only available structure elucidating direct interaction between an ion channel and a Gα subunit is that of TRP canonical (TRPC) 5 and Gα_i3_^79^ (Fig. 3a). This structure provides a representative example of how Gα binds to a TRP channel to modulate its function, raising intriguing questions about the broader role of Gα in ion channel regulation.

### TRPC5–Gα_i3_ complex

TRPC5 is a calcium-permeable, nonselective cation channel, primarily expressed in the brain. It exhibits constitutive activity, which can be further enhanced by various stimuli, such as intracellular calcium^80^, oxidation^81^ and acidification^82^. In addition, TRPC5 plays a key role in cold detection within the physiological temperature range (25–37 °C) in the peripheral nervous system, functioning as a calcium-permeable channel^83^. The channel shows a unique doubly rectifying current–voltage curve, which is notably different from its heteromeric configuration with TRPC1, suggesting a distinct mode of channel activity compared to other ion channels^84^.

The direct activation of TRPC5 by Gα_i_ has been extensively studied in both the gastrointestinal tract and overexpression systems^85–87^. A recent cryo-EM structure of TRPC5 revealed its complex with the activator Gα_i3_, which binds to the channel’s cytosolic ankyrin repeat domain (ARD)^79^ (Fig. 3b). As the role of the ARD in TRPC5 regulation remains incompletely understood, this structure raises several intriguing questions. One particularly interesting aspect regarding Gα_i3_ binding to the ARD is how Gα_i3_ can bind to a site on the ARD that is ~50 Å away from the plasma membrane. A potential explanation is found in earlier studies showing that the N-terminal α-helix of inactive Gα unfolds upon GDP-to-GTP exchange^88^. This suggests that the N terminus of GTP-bound Gα_i3_ could unfold, extending far enough from the membrane to allow Gα_i3_ to bind to the ankyrin repeat edge of the TRPC5 channel. Simultaneously, the N-terminal lipidation of Gα_i3_ keeps it tethered to the membrane, facilitating its regulatory interaction with TRPC5. A similar distance (~40 Å) between the effector-binding site of Gα and the membrane has been observed for AC^42^, supporting the idea that the flexible N terminus of activated Gα expands its range of interactions with protein effectors.

Another crucial question is how Gα_i3_ binding to the ARD domain, which is located 50–54 Å away from the lower gate, contributes to channel gating. To fully elucidate the underlying mechanism, the open-state structure of the TRPC5–Gα_i3_ complex would be required. However, the overall architecture of TRPC5 in the TRPC5–Gα_i3_ complex closely resembles previously reported structures of tetrameric TRPC5 in the closed state^79^. So far, no open conformation has been resolved, leaving many aspects of its gating mechanism and regulation unexplored.

To take a closer look at the binding interface between TRPC5 and Gα_i3_, a cavity formed by the α2 and α3 helices of Gα_i3_ interacts with the ARD 1–2 regions of TRPC5. Specifically, the IYY motif of TRPC5, consisting of residues I57, Y58 and Y59, interacts with Gα_i3_’s residues R205, R208 and E245, as well as W211, I212 and F215 (Fig. 3c). Notably, this IYY motif in TRPC5 is not conserved across the TRPC subfamily, while leaving TRPC4 as the probable exception, which possesses the ability to be directly potentiated by Gα_i_^87^. The binding of Gα_i3_ to TRPC5 also appears to be guided by long-range electrostatic interactions near switch II. The strongly electronegative surface of the ARD 1–2 regions of TRPC5 is hypothesized to attract the electropositive binding groove of Gα_i3_, thereby facilitating its approach to the channel. Of note, similar to observations in the GIRK2–Gβγ complex, positive cooperativity was detected in inside-out patches of TRPC5 with Gα_i3_, as indicated by a calculated Hill coefficient (*n*) of ~2.3 (ref. ^79^).

Although the open-state structure of TRPC5 upon Gα binding has not yet been resolved, it is noteworthy that the cytosolic domain of TRPC5 adopts two distinct conformations, referred to as class 1 and class 2. These conformational states involve an 8° rotation of the ARD and a 45° rotation of the coiled-coil domain (CCD) (Fig. 3d). While the precise functional significance of these conformations remains unclear, two potential hypotheses can be proposed. First, in most TRP channels where the open-state structure has been determined, conformational changes in the cytosolic domain are associated with central pore gating^89–93^. This suggests that Gα_i3_ binding to the ARD may indirectly influence the pore region formed by the S5–S6 helices, thereby facilitating channel activation. Second, structural insights from TRPC3 and TRPC6 indicate that an ion-permeable pore exists between the ARD and CCD, where calcium ions bind and inhibit channel activity^94^. Similarly, the transition between class 1 and class 2 in TRPC5 may induce dilation between the ARD and CCD, potentially influencing channel function. On this basis, it can be hypothesized that Gα_i3_ binding promotes pore dilation within the cytosolic domain, thereby enhancing TRPC5 activity. However, further studies are required to validate this hypothesis.

Another possible mechanism underlying TRPC5 activation by Gα_i3_ is its indirect role in facilitating channel opening by modulating the activity of PIP_2_, another agonistic molecule for TRPC5. Electrophysiological data suggest that Gα_i3_ can directly activate TRPC5 by increasing its sensitivity to PIP_2_, by lowering the dissociation constant of PIP_2_ toward TRPC5^79^. Notably, in the absence of PIP_2_, Gα_i3_-mediated channel activation is completely abolished^79^.

### The binding of Gα to other ion channels

The direct binding of Gα_i_ has been extensively studied not only in TRPC5 but also in the TRPC1/4/5 group. Our research group, along with others, previously demonstrated that Gα_i2_ directly binds to and activates TRPC4, probably in a manner similar to TRPC5 (refs. ^87,95^). However, this activation is abolished in TRPC heteromers containing TRPC1 (TRPC1/C4 and TRPC1/C5)^96^, although the underlying mechanism remains unclear. For the TRPC1/C4 heteromer, a recently published cryo-EM structure with a 1:3 stoichiometry reveals that the IYY motif—essential for Gα_i_ activation in TRPC5—is absent in both TRPC1 and the TRPC4 subunit^97^. This structural difference may explain the loss of Gα_i_ binding and subsequent activation in the heteromer.

Contrary to Gα_i_, Gα_q_ has been proposed to activate heteromeric TRPC1/C4 and TRPC1/C5 channels, despite the lack of structural evidence supporting this interaction^98^. Gα_q_, activated by the inflammatory bradykinin 2 GPCR, is suggested to directly bind to TRPM8 and inhibit its activity—bypassing the PLCβ-mediated PIP_2_ depletion mechanism that is commonly associated with channel inhibition^99,100^. Structural studies of TRPC1/C4, TRPC1/C5 or TRPM8 in complex with Gα_q_ would provide crucial insights into the bidirectional regulation of different TRP channels by Gα_q_. Activation of TRPC1 by direct binding of Gα_11_ has been consistently reported by multiple groups^101,102^. In addition, while the individual or combined effects of Gα_o_ and Gβγ remain unclear, the binding of Gα_o_ to TRPM1 has been demonstrated through co-immunoprecipitation and bioluminescence resonance energy transfer assays^72,75^, classifying TRPM1 as a Gα-binding ion channel.

Beyond the TRP channel family, several ion channels have also been shown to be directly regulated by Gα. For instance, Gα_o_ directly activates K^+^ channels in the brain^103,104^, whereas Gα_s_ both activates cardiac Ca^2+^ channels^105–107^ and inhibits cardiac Na^+^ channels^108^ through direct interactions. In most cases, the GTP-bound active state of Gα is essential for its binding and regulatory function. Upon GTP binding, structural changes occur in the switch I–III regions of the Ras-like GTPase domain^109^ (Fig. 3e). Among these, switch II plays a key role in interactions with downstream effectors and also acts as a trigger for the dissociation of Gβγ from the heterotrimer. The α2-helix of switch II has been identified as a major effector-binding motif in several complex structures, including those with Gβγ, ACs, PLCβ, RGS and the TRPC5 channel (Fig. 3f). This region serves as a critical ‘hotspot’ for protein–protein interactions, facilitating the direct regulation of ion channels.

## Direct binding of small G proteins to ion channels

Small G proteins containing a GTP-binding domain have recently been shown to directly regulate TRP channels, particularly TRP vanilloid 4 (TRPV4)^16–18^ (Fig. 4a). Although further studies are warranted, structural analyses have revealed that TRPV4 remains constitutively bound to RhoA in its closed state when recombinantly expressed. The recognition of this novel binding mode by multiple independent groups has deepened our understanding of the inter-regulation between ion channels and small GTPases while also shedding light on disease-associated mutations in TRPV4.

### TRPV4–RhoA complex

The involvement of TRP channels in cell migration has been well studied^110,111^, and given the critical role of Rho-like small GTPases in this process, the interaction between TRP channels and the Rho-like family has been investigated^112^. Recently, cryo-EM structures of the TRPV4–RhoA complex have been elucidated by three independent groups, revealing that the β1, β3, switch I and switch II regions of RhoA interact with AR2–AR5 of each N terminus from the four TRPV4 subunits, thereby inhibiting the channel’s activity^16–18^ (Fig. 4a,b). Notably, these binding sites are unique to both RhoA and TRPV4 and are not shared with other isoforms of either protein, highlighting the specificity of this direct interaction.

In the agonist-induced open conformation (PDB, 8FCB), the ARD skirt of TRPV4 rises toward the lipid bilayer, while the electron density of RhoA weakens, and the interaction distance between E40 and N41 (RhoA) and R224 (TRPV4) increases from 2.7–2.8 Å to approximately 4.3–4.5 Å (Fig. 4c). As a result, the smallest binding interface is observed in the open state of the TRPV4–RhoA complex (Fig. 4a). These findings suggest that RhoA acts as a tonic inhibitor of TRPV4 by mechanically restricting the upward movement of the ARD region^16^. This aligns well with previous studies indicating that, during channel opening, RhoA dissociates from TRPV4, subsequently influencing cytoskeletal dynamics^113,114^.

### Regulatory role of small G protein-binding to ion channels

Contrary to the regulatory role of RhoA in TRPV4 channel function, other small G proteins contribute to vesicle trafficking and cell migration by binding to TRP channels. One of the key regulators of intracellular vesicle trafficking, Rab11a, binds to the C termini of TRPV5 and TRPV6 vesicles in its GDP-bound form, thereby controlling membrane trafficking^115^. Upon activation, Rab11a dissociates from the ion channel but remains associated with other effectors within the vesicle. This interaction facilitates vesicle movement toward the plasma membrane, ensuring precise trafficking and channel delivery.

In addition, TRP channels also regulate small G proteins through direct interactions. For example, TRPC5 has been shown to activate Rac1, while TRPC6 activates RhoA through direct binding^116^. TRPM2 directly interacts with Rac1 and activates it, although the exact mechanism remains unclear^117^. In turn, Rac1 enhances the membrane trafficking of TRPM2, forming a positive feedback loop that exacerbates ischemic injury^117^. Furthermore, functional studies using site-directed mutagenesis have revealed that the TRPM8 channel in the endoplasmic reticulum captures inactive Rap1A from the cytosol, leading to the inhibition of endothelial cell migration^118^.

Beyond TRP channels, small G proteins also directly regulate other ion channels, such as the regulation of RGK on Ca_V_ channels^119,120^. RGK is a subfamily within the Ras superfamily, consisting of four members: Rad, Rem, Rem2 and Gem/Kir^121^. All RGK proteins directly inhibit high-voltage-activated calcium channels (Ca_V_1 and Ca_V_2)^120–124^. Unlike Ras, RGKs contain distinct sequences, with their C termini playing a key role in inhibiting Ca_V_ channels^123,125–127^. Overall, an increasing number of functional roles for small G proteins in ion channel regulation have been identified, highlighting the need for further structural studies to elucidate these mechanisms.

## Concluding remarks

GPCRs and ion channels, two biologically and pharmaceutically important classes of membrane proteins, exhibit intricate crosstalk. Indirect crosstalk occurs through small secondary messengers, where GPCR activation triggers downstream signaling pathways that modulate ion channel activity. Alternatively, direct binding of Gα or Gβγ subunits to ion channels can directly regulate their ion conduction. Increasing evidence has revealed direct interactions between Gα or Gβγ subunits and ion channels, highlighting their crucial functional roles. To gain a mechanistic understanding of ion channel activation through Gα or Gβγ binding, structural studies are essential. These studies will provide insight into the precise binding sites of Gα or Gβγ subunits on ion channels and how these interactions alter ion channel conformations. In recent years, cryo-EM structures of ion channels complexed with G proteins have been resolved, offering valuable structural insights into these regulatory mechanisms.

A deeper mechanistic understanding of how G proteins directly modulate ion channels could also provide critical insights into channelopathies, which may arise from disruptions in G protein binding. Interestingly, current structural and biochemical studies suggest that, in many cases, the N-terminal region of ion channels is involved in G protein binding. Consistently, many disease-associated mutations linked to channelopathies or syndromes are localized in this region, as shown in Fig. 4f and Table 3. Identifying the exact binding sites of G proteins on ion channels could offer potential drug targets for modulating protein–protein interactions between G proteins and ion channels.

**Table 3:** Disease-related mutations in the N-terminal region of TRP channels.

Channel gene	Reported G protein interaction	PDB/AlphaFold Code^a^	Structural domain^b^	Disease-related mutation^c^	OMIM code
TRPV4	RhoA^16–18,113,114^	8FC9 (cryo-EM)	Far N terminus	P19S, G78W, T89I	605427
			Inner ARD	K197R, F273L, K276E, I331F, D333G	
			ARD surface	**E183K**, R186Q, **R232C,** **R269H/C**, G270V, R271P, E278K, **R315W,** **R316H/C**	
TRPM1	Gβγ, Gα_o_^72,75^	Q7Z4N2 (AlphaFold)	Far N -terminus	Q11T	603576
			Inner MHR	L99P, R624C	
TRPM3	Gβγ^51,52^	8DDW (cryo-EM)	Far N terminus	I65M	608961
TRPM4	–	6BQV (cryo-EM)	Far N terminus	E7K	606936
			Inner MHR	A432T	
			MHR surface	R164W	
TRPM6	–	Q9BX84 (AlphaFold)	Inner MHR	S141L	607009

^a^If the structure of the channel is revealed by cryo-EM, its PDB ID is provided. Otherwise, the AlphaFold code of the channel is provided.

^b^In the case of residues for which structural information has not been revealed owing to their location in a flexible region, their structural domain is classified as ‘Far N terminus’. In a three-dimensional model, if a residue is facing outward from the protein, it is classified as being on the ‘surface’; otherwise, it is classified as being in the inner ARD or MHR.

^c^Amino acid mutations documented to directly interact with G proteins are shown in bold.

Previous structural studies on G protein–ion channel interactions have markedly advanced our understanding of direct regulation; however, several key questions remain unresolved: (1) How does G protein binding, particularly Gα, trigger conformational changes in ion channels? (2) Is there potential cooperativity among Gα or Gβγ subunits bound to ion channels? (3) What is the stoichiometry between Gα (or Gβγ) and ion channels? (4) How does Gα (or Gβγ) binding interact with other ion channel modulators, such as PIP_2_? (5) Is there a potential for the formation of a GPCR–ion channel megacomplex? Further structural and functional studies will be essential to addressing these questions and elucidating the fundamental mechanisms underlying G protein–ion channel interactions.
